# High-resolution MRI data of the brain of C57BL/6J and BTBR mice in three anatomical views

**DOI:** 10.1016/j.dib.2021.107619

**Published:** 2021-11-20

**Authors:** Yulia A. Ryabushkina, Oleg B. Shevelev, Polina E. Kisaretova, Nikita G. Sozonov, Kseniya A. Ayriyants, Natalya P. Bondar, Vasiliy V. Reshetnikov

**Affiliations:** a Institute of Cytology and Genetics, Siberian Branch of Russian Academy of Sciences (SB RAS), Prospekt Lavrentyeva 10, Novosibirsk 630090, Russia; b Novosibirsk State University, Pirogova Street 2, Novosibirsk 630090, Russia; c Sirius University of Science and Technology, 1 Olympic Avenue, Sochi 354340, Russia

**Keywords:** BTBR, C57BL/6J, Early-life stress, MRI, Brain anatomy, Strain-specific, Sex-specific

## Abstract

The research on strain-, sex-, and stress-specific differences in structural and functional connectivity of the brain is important for elucidating various behavioral features and etiologies of psychiatric disorders. Socially impaired BTBR mice are considered a model of autism spectrum disorders. Here we present high-resolution magnetic resonance imaging data from the brain of 89 adolescent mice (C57BL/6J and BTBR) in axial, sagittal, and coronal views. The study [1] includes both females and males differed in early-life experience (normally reared or subjected to prolonged maternal separation: 3 h daily from postnatal day 2 to 15). The MRI data were obtained on a horizontal tomograph Biospec 117/16 instrument with a magnetic field strength of 11.7 T. Thus, multislice Turbo RARE T_2_-weighted images of the brain were captured in eight groups of mice. Altogether, these data allow to evaluate strain-, sex-, and stress-specific alterations in the volumes of various brain structures and to better understand the relation between brain structural differences and behavioral abnormalities.

## Specifications Table

**Table:** 

Subject	Neuroimaging
Specific subject area	Small-animal brain magnetic resonance imaging
Type of data	Image, Table
How data were acquired	Images were acquired on a horizontal tomograph (Biospec 117/16 USR, Bruker, Germany) with a magnetic field strength of 11.7 T. Cranial, sagittal, and axial scans (section thickness, 0.5 mm; field of view, 2.5 × 2.5 mm; matrix, 256 × 256 dots) were performed on all mice.
Data format	Raw
Parameters for data collection	In vivo MRI of the brain of adolescent mice was carried out in three anatomical views. Eight groups of mice were used in the study: Control (normally reared) C57BL/6J males Control (normally reared) C57BL/6J females Control (normally reared) BTBR males Control (normally reared) BTBR females Stressed C57BL/6J males (maternal separation) Stressed C57BL/6J females (maternal separation) Stressed BTBR males (maternal separation) Stressed BTBR females (maternal separation) As an experimental model of postnatal stress, the separation of pups from dams was performed daily for 3 h from postnatal day (PND) 2 to PND15 (the day of birth was designated as PND0). MRI was carried out intravitally on PND40 when the animals were immobilized by isoflurane and then restrained by the paws on an MRI table.
Description of data collection	The scans of the brain were performed by means of a receive-transmitter volume (T12970V3) 1H radiofrequency coil of a horizontal tomograph with a magnetic field of 11.7 T (Bruker, Biospec 117/16 USR, Germany). T_2_ images in axial, sagittal, and coronal projections were captured by the rapid with relaxation enhancement (Turbo RARE) method with the following pulse sequence parameters: TE = 11 ms, TR = 2.5 s (slice thickness 0.5 mm, field of view 2.0 × 2.0 cm, and matrix size 256 × 256 pixels). From each mouse, on average, 29 axial, 20 sagittal, and 15 coronal scans were obtained.
Data source location	Institute of Cytology and Genetics, SB RAS, Prospekt Lavrentyeva 10, Novosibirsk, Russian Federation
Data accessibility	The raw data of MRI were deposited in the Mendeley Data repository doi: https://doi.org/10.17632/dz9x23fttt.1 https://data.mendeley.com/datasets/dz9x23fttt/1
Related research article	Vasiliy V. Reshetnikov, Kseniya A. Ayriyants, Yulia A. Ryabushkina, Nikita G. Sozonov, Natalya P. Bondar, Sex-specific behavioral and structural alterations caused by early-life stress in C57BL/6 and BTBR mice, Behavioural Brain Research, Volume 414, 2021, 113489, ISSN 0166-4328, https://doi.org/10.1016/j.bbr.2021.113489[1]

## Value of the Data

• The data enable the assessment of stress-, sex-, and strain-specific differences in the brain of C57BL/6J and BTBR mice
• These data can be employed by neuroscience researchers for evaluating the influence of early-life stress on structural parameters of the brain in mice of different strains
• The data can be used in a subsequent analysis for collating the brain differences in BTBR mice with other models of neurodevelopmental disorders

## 1 Data Description

**Fig. 1:**
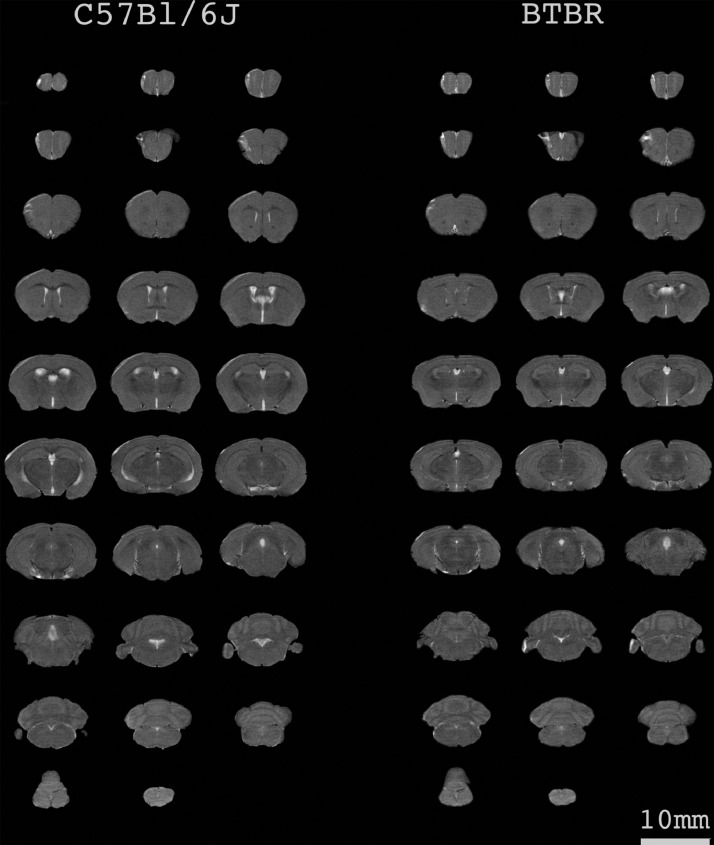
Representative MRI scans of C57BL/6J and BTBR mice in the axial projection. Twenty-nine consecutive MRI scans in the dorsal-ventral orientation in a male mouse chosen randomly for the figure.

**Fig. 2:**
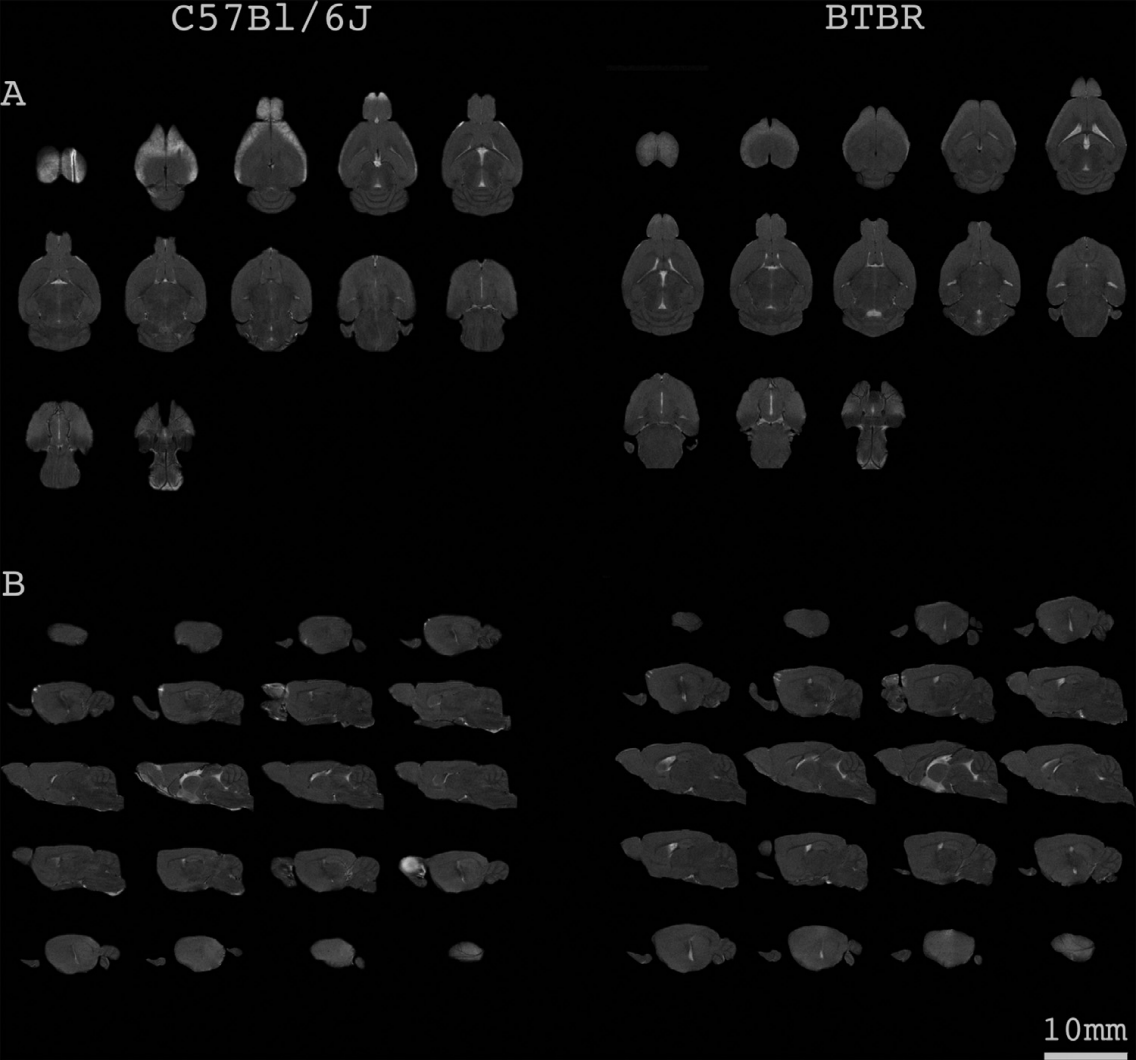
Representative MRI scans of C57BL/6J and BTBR mice in coronal and sagittal projections **a.** Twelve to 13 consecutive MRI scans in the caudal-cranial orientation in a male mouse chosen randomly for the figure **b.** Twenty consecutive MRI scans in the medial-lateral orientation in a male mouse chosen randomly for the figure.

These data are intravital serial high-resolution magnetic resonance imaging (MRI) scans of the brain in female and male mice of strains C57BL/6J and BTBR in three anatomical views. Eight groups of mice were used in the study (Table 1). The MRI was conducted at age 40 days (PND40). Each group consisted of 8 to 16 animals, and in total, 89 mice were analyzed. On average, in each animal, 29 axial, 20 sagittal, and 12 coronal slices were analyzed. Examples of MRI scans from C57BL/6J and BTBR mice in different anatomical views are presented in Figs. 1 and 2. The data allow for the evaluation of stress-, sex-, and strain-specific volumetric and structural differences in the brain of C57BL/6J and BTBR mice and should help to determine detailed 3D structure of the brain.

**Table 1:** MRI data summary.

Strain	Group	Sex	Age	Number of mice	Average number of axial slices	Average number of sagittal slices	Average number of coronal slices
BTBR	Control	Male	PND40	13	29	20	13
BTBR	Control	Female	PND40	11	29	20	13
BTBR	Maternal separation	Male	PND40	16	29	20	13
BTBR	Maternal separation	Female	PND40	8	29	20	12
C57BL/6	Control	Male	PND40	10	31.9	22	12
C57BL/6	Control	Female	PND40	10	31.9	22	12
C57BL/6	Maternal separation	Male	PND40	12	31.9	20	12
C57BL/6	Maternal separation	Female	PND40	9	31.9	20	12

## 2 Experimental Design, Materials and Methods

### 2.1 Mice and housing

The study was conducted at the multi-access center “SPF-vivarium” of the Institute of Cytology and Genetics, SB RAS (project identifier: RFMEFI62119 × 0023). C57BL/6J and BTBR T + Itpr3tf/J (BTBR) mice were purchased from Jackson Laboratory and were kept under standard conditions of a specific pathogen-free (SPF) animal facility in triangular cages with dimensions 34.3 cm (L) × 29.2 cm (W) × 15.5 cm (H) (Optimice, Animal Care Systems, Inc.) and containing bedding (birch chips) and nesting material. Food (pellets) and water were available *ad libitum*.

### 2.2 Maternal-separation procedures

Litters C57BL/6J and BTBR mice were randomly assigned to either a maternal separation group or a control (normal rearing conditions) group. Each litter consisted of 5 to 8 animals. Pups in the stress group were subjected to prolonged separation from their mothers (3 h once a day) from PND2 to PND15 as described elsewhere [1,2]. Briefly, each pup was placed in an individual plastic box (11 × 7 cm) filled with bedding, whereas the dam stayed in the home cage during the separation procedure. This type of maternal separation prevents contact between siblings during the separation procedure. During the separation, the temperature was kept at 30°C ± 2°C using a heat mat to prevent thermoregulatory distress. On PND21, the pups of both groups were weaned and placed into separate cages in groups of 2–3 siblings of the same sex.

### 2.3 Experimental design

Eight groups of mice were set up: control C57BL/6J males and females, control BTBR males and females, maternally separated C57BL/6J males and females, and maternally separated BTBR males and females. On PND37–38, the mice were subjected to behavioral tests as described previously [1]. On PND40, these animals underwent the intravital volumetric analysis of various brain regions by MRI.

### 2.4 MRI

Intravital morphological analyses of the brain were conducted on a horizontal tomograph with a magnetic field strength of 11.7 Tesla (Biospec 117/16; Bruker, Billerica, MA, USA). Three minutes prior to the scanning, the animals were immobilized by gas anesthesia (Isofluran, Baxter International Inc.) using an anesthesia machine (Univentor 400 Anaesthesia Unit; Univentor Ltd., Zejtun, Malta). The immobilization was performed using 4.2% isoflurane at an airflow rate of 250 ml/min and was maintained with 1.8% isoflurane at the same flow rate. The body temperature of the mice was maintained by means of a water circuit within a tomographic bed-table with a surface temperature of 35°C. A pneumatic breathing sensor (SA Instruments Inc., Stony Brook, NY, USA) was placed under the lower torso and made it possible to control the depth of the anesthesia.

All images of mouse brains were acquired using a receive-transmitter volume (T12970V3) 1H radiofrequency coil. T_2_ images in the three anatomical views were captured by the rapid with relaxation enhancement (Turbo RARE) method with the following pulse sequence parameters: TE = 11 ms, TR = 2.5 s, and RARE factor = 4 (slice thickness 0.5 mm, field of view 2.0 × 2.0 cm, and matrix size 256 × 256 pixels). The total scan time was 20 min per animal. All the images were exported as dicom files in the ParaVision 5.1 Software (Bruker).

## Ethical Statement

Male and female C57BL/6 and BTBR mice were maintained at the Animal Facility of the Institute of Cytology and Genetics, SB RAS, Novosibirsk, Russia (RFMEFI62119 × 0023). All the procedures were approved by the Ethical Committee at the Institute of Cytology and Genetics, SB RAS (Protocol #25, December 2014) in conformity with EU Directive 2010/63/EU for animal experiments.

## CRediT authorship contribution statement

**Yulia A. Ryabushkina:** Conceptualization, Methodology, Writing – original draft. **Oleg B. Shevelev:** Investigation, Data curation. **Polina E. Kisaretova:** Investigation, Data curation. **Nikita G. Sozonov:** Investigation, Data curation. **Kseniya A. Ayriyants:** Investigation, Data curation. **Natalya P. Bondar:** Supervision, Writing – review & editing. **Vasiliy V. Reshetnikov:** Conceptualization, Methodology, Writing – original draft.

## Declaration of Competing Interest

None.
